# T2/FLAIR Abnormity Could be the Sign of Glioblastoma Dissemination

**DOI:** 10.3389/fneur.2022.819216

**Published:** 2022-02-02

**Authors:** Mingxiao Li, Wei Huang, Hongyan Chen, Haihui Jiang, Chuanwei Yang, Shaoping Shen, Yong Cui, Gehong Dong, Xiaohui Ren, Song Lin

**Affiliations:** ^1^Department of Neurosurgical Oncology, Beijing Tiantan Hospital, Capital Medical University, Beijing, China; ^2^Department of Neurosurgery, Beijing Neurosurgical Institute, Capital Medical University, Beijing, China; ^3^Department of Radiology, Beijing Tiantan Hospital, Capital Medical University, Beijing, China; ^4^Department of Neurosurgery, Peking University Third Hospital, Peking University, Beijing, China; ^5^Department of Pathology, Beijing Tiantan Hospital, Capital Medical University, Beijing, China; ^6^Department of Neuroscience, Beijing Key Laboratory of Brain Tumor, Institute for Brain Disorders, Center of Brain Tumor, Beijing, China

**Keywords:** glioblastoma (GBM), isocitrate dehydrogenase (IDH), gross total removal of tumor (GTR), supratotal maximal resection (SMR), T2/FLAIR, progression, dissemination, RANO

## Abstract

**Purpose:**

Newly emerged or constantly enlarged contrast-enhancing (CE) lesions were the necessary signs for the diagnosis of glioblastoma (GBM) progression. This study aimed to investigate whether the T2-weighted-Fluid-Attenuated Inversion Recovery (T2/FLAIR) abnormal transformation could predict and assess progression for GBMs, especially for tumor dissemination.

**Methods:**

A consecutive cohort of 246 GBM patients with regular follow-up and sufficient radiological data was included in this study. The series of T2/FLAIR and T1CE images were retrospectively reviewed. The patients were separated into T2/FLAIR and T1CE discordant and accordant subgroups based on the initial progression images.

**Results:**

A total of 170 qualified patients were finally analyzed. The incidence of discordant T2/FLAIR and T1CE images was 25.9% (44/170). The median time-span of T2/FLAIR indicated tumor progression was 119.5 days (ranging from 57 days-unreached) prior to T1CE. Nearly half of patients (20/44, 45.5%) in the discordant subgroup suffered from tumor dissemination, substantially higher than accordant patients (23/126, 20.6%, *p* < 0.001). The median time to progression (TTP), post-progression survival (PPS), and overall survival (OS) were not statistically different (all *p* > 0.05) between discordant and accordant patients.

**Conclusions:**

T2/FLAIR abnormity could be the sign of GBM progression, especially for newly emerged lesions disseminating from the primary cavity. Physicians should cast more attention on the dynamic change of T2/FLAIR images, which might be of great significance for progression assessment and subsequent clinical decision-making.

## Introduction

The glioblastoma (GBM) is one of the most lethal malignancies and harbors profoundly intratumoral and intertumoral heterogeneity (1–3). This heterogeneity encompasses substantially molecular and spatial-temporal distinction and could be reflected on imaging (4). Though GBM typically presents as contrast-enhancing tumors (CET) on MRI, components beyond CE margins, regarded as non-CE tumors (nCET), could also progress rapidly and evolve to CET that severely threaten survival (5). Recently, dozens of studies focusing on the nCET have proposed the innovative surgical strategy that nCET should be considered to be removed, which might be helpful to prolong prognosis (6–8). This informed us that more attention should be attached to the nCET.

Multicentric and multifocal GBM, consisting of 1–35% newly diagnosed GBM, tend to portend a worse prognosis than unifocal (9). In contrast to multicentric GBM, multifocal GBM presents obvious communication on T2-weighted-Fluid-Attenuated Inversion Recovery (T2/FLAIR) imaging. However, both the definition of multicentric and multifocal GBMs require CE rather than the non-CE (nCE) lesions as one of the centers or focuses (10). Till now, few researchers reported GBMs with nCE lesions as multi-focal or multicentric GBMs. Lasocki et al. firstly reported that nine (6%) of 151 patients with GBM had isolated nCE lesions and further proved GBM, which warned us to pay more attention to this phenomenon (11).

Multifocal or multicentric lesions could not only be diagnosed for primary GBM but for recurrent or progressed tumors. Though local recurrence dominates the patterns of progression, non-local progression, such as distant intracranial metastasis, subependymal spread, and leptomeningeal dissemination, and extracranial visceral metastasis, occurs in 2–34.5% patients with GBM (12, 13). The Response Assessment in Neuro-Oncology (RANO) and modified RANO criteria warrant new emerged or significantly enlarged CE lesions as the necessary and essential evidence to consider tumor progression for Bevacizumab-naïve patients (14–16), nevertheless, non-local recurrence could be similar to nCE multifocal or multicentric GBMs. They might not be visible on T1CE but T2/FLAIR images. Till now, no studies focused on this issue. Thus, we performed a retrospective study to explore whether T2/FLAIR could be more sensitive in distinguishing early progression than T1CE images, especially for non-local progressed GBM.

## Methods and Materials

### Patients

A cohort of 246 consecutive adult patients from March 1, 2013 to August 31, 2020, surgically treated and pathologically defined as *de novo* supratentorial isocitrate dehydrogenase (IDH) wild-type GBM based on 2021 WHO classification of brain tumors was included in this retrospectively study (17). All tissue sections were meticulously reviewed by 3 senior neuropathologists to generate a consensus diagnosis. Patients with inadequate follow-up, lethal comorbidity, or other malignancies were excluded. Besides, patients without tumor progression were not included for subsequent analysis. Clinical, radiological, and pathological information was recorded.

### Molecular Information

The 1p/19q codeletion, 7+/10–, epidermal growth factor receptor (EGFR) amplification status were determined by fluorescence *in situ* hybridization (FISH). For IDH1 R132 and IDH2 R172 mutations, telomerase reverse transcriptase (TERT) promoter C228T/C250T mutation was tested by Sanger sequencing (18, 19). The status of O^6^-methylguanine-DNA methyltransferase (MGMT) promoter was determined by pyrosequencing, and patients were divided into methylated and unmethylated by the average methylation level of 12% (20). BRAF V600E, fibroblast growth factor receptor 1 (FGFR1), and H3K27M mutations were evaluated by Sanger sequencing for exclusion when required.

### Collection of Radiological Data

All MRI studies were performed on 3.0-T clinical scanners (Siemens Trio Tim, or GE, Boston, MA USA) in the routine clinical workup. The protocol included axial T1-weighted (repetition time [TR] 1,750–2,250 ms, echo time [TE] 9.4–19.8 ms, matrix 256 × 198, slice thickness 5 mm), T2-weighted fast spin-echo (TR 4,900–6,711 ms, TE 97–116.6 ms, FA = 150°, matrix 256 × 320, slice thickness 5 mm, spacing 1 mm, field of view [FOV] = 220 × 220 mm, number of excitations [NEX] = 3), T2 FLAIR (TR = 7,000–8,000 msec, TE 91–152.0 msec, TI 2,340 ms, matrix 256 × 186; slice thickness 5 mm, spacing 1 mm, FOV = 220 × 220 mm, NEX = 3), and axial and coronal contrast-enhanced T1-weighted images (CE-T1WI; TR 1,779.2–2,110 ms, TE 9.4–19.8 ms, matrix: 320 × 288, FA = 15°, FOV = 240 × 188 mm, slice thickness 5 mm, spacing 1 mm, NEX = 1) with the administration of gadopentetate dimeglumine (0.2 mmol/kg).

MRI examinations were independently analyzed by 2 investigators (XHR, a neurosurgical oncologist with 15 years of experience and HYC, a radiologist with 25 years of experience). Both were blinded to clinical history, molecular status, and histopathologic diagnosis. Reassessment was performed when discordant results were acquired. If the disagreement persisted, a third reviewer (XZC, a radiologist in brain imaging with 25 years of experience), joined the discussion for final consensus.

### Treatment and Follow-Up

All enrolled patients were surgically treated. After the operation and a waiting period of about 3–5 weeks, the Stupp's protocol, radiation with guideline-recommended dose concurrent daily temozolomide (TMZ; 75 mg/m^2^/d), was finished, and following cycles of maintenance TMZ (150–200 mg/m^2^ for 5 days every 28 days) adjuvant chemotherapy was administered.

Contrast-enhanced-MRI was meticulously followed within 4 weeks after concurrent chemoradiotherapy (CCRT) and regularly surveilled with an interval of 8–12 weeks or if necessary. The patterns of tumor progression were classified as local or *in situ* (obvious connection with the primary resection cavity), distant intracranial metastasis (newly emerged parenchyma lesions without a clear connection with the original tumor on T2/FLAIR images), subependymal spread (lesions disseminated along with the subependymal zone), and leptomeningeal dissemination (diffuse leptomeningeal enhancement around the contours of the gyri and sulci with/without multiple nodular deposited in the subarachnoid space) based on the initial MR images with progression (12). We also employed the principles of RANO for low-grade glioma to evaluate the dynamic change of T2/FLAIR of this GBM cohort (21). MR spectrum (MRS), perfusion-weighted MRI (PWI) by dynamic susceptibility contrast (DSC), and ^18^F-FDG-PET MRI were available to some but not all patients during the follow-up to provide valuable information to distinguish treatment response (pseudoprogression and radiation necrosis) from true progression.

To distinguish peritumor edema and true non-enhancing tumor, we introduced these definitions from the Visually Accessible Rembrandt Images (VASARI) feature set (https://wiki.nci.nih.gov/display/CIP/VASARI). Edema should be greater in signal than nCET and somewhat lower in signal than CSF. Pseudopods are the canonical characteristics of edema. The entire abnormality may be comprised of: (1) an enhancing component, (2) a non-enhancing component, (3) a necrotic component, and (4) an edema component for a typical GBM.

Time to progression (TTP) was defined as the duration from the initial surgery to the time of true tumor progression, and overall survival (OS) was termed as the duration between the initial surgery and the death, or date of the last follow-up (19, 22). Post-progression survival (the time span between tumor progression and death) was also calculated and documented for further analysis. All assessments were performed prior to Bevacizumab or other antiangiogenic therapy.

### Statistical Analysis

The student's *t*-test was used for continuous variables, and the Mann-Whitney *U*-test was applied for non-parametric data. The Chi-square test or Fisher's exact test was used to compare the categorical variables. Graphpad Prism (Version 8.0.1, GraphPad Software, San Diego, CA, USA) was used for statistical analysis. The survival rate of patients was estimated with the Kaplan-Meier plot, and differences between curves were compared by the log-rank test. Probability values were obtained using 2-sided tests with statistical significance defined as *p* < 0.05.

## Results

### Descriptive Characteristics and the Incidence of Different Patterns of Progression

**Figure 1:**
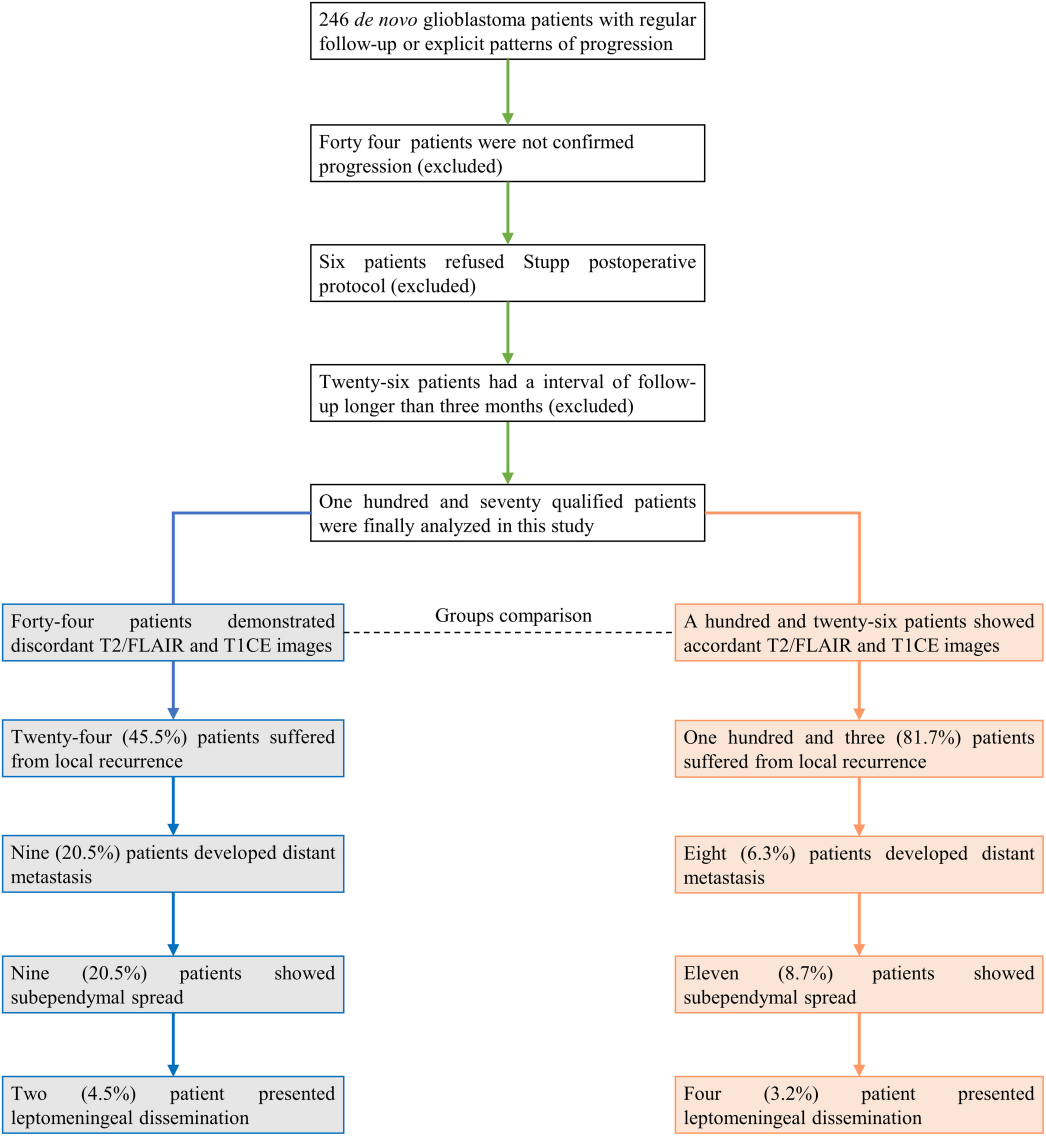
Patients included in this study. The final analysis showed that 25.9% of patients (44/170) presented discordant results between T2/FLAIR and T1CE images.

A total of 246 patients were initially included in this study. Patients with no recurrence, ambiguous diagnosis of progression, or follow-up interval longer than 3 months were excluded for subsequent analysis (Figure 1). In 170 qualified GBM patients with assessable progression patterns, 25.3% (43/170) demonstrated non-local progression, such as distant intracranial metastasis (17/170, 10.0%), subependymal spread (20/170, 11.8%), and leptomeningeal dissemination (6/170, 3.5%), while local or diffuse recurrence was present in the other 127 (74.7%) patients (Table 1).

**Table 1:** Clinical, demographic and radiological characteristics of patients with/without discordant T2/FLAIR T1CE images.

**Characteristics**	**Discordant**	**Accordant**	***P*-value**
Number of patients	44 (25.9%)	126 (74.1%)	-
Age at diagnosis (years)			
Mean	46.3 ± 12.8	49.6 ± 12.1	0.130
Median	50.0	51.0	0.847
Gender			
Male	30 (68.2%)	85 (67.5%)	0.930
Female	14 (31.8%)	41 (32.5%)	
Preoperative KPS			
>70	36 (81.8%)	81 (64.3%)	**0.031**
≤ 70	8 (18.2%)	45 (35.7%)	
Extent of resection			
GTR	32 (72.7%)	64 (50.8%)	**0.012**
Non-GTR	12 (27.3%)	62 (49.2%)	
Tumor volume (cm^3^)			
Mean	36.8 ± 33.1	44.5 ± 51.7	0.352
Median	31.7	38.0	0.330
Ventricle infringement			
Yes	23 (52.3%)	79 (62.7%)	0.224
No	21 (47.7%)	47 (37.3%)	
MGMT promoter			
Methylated	19 (43.2%)	44 (35.8%)	0.384
Unmethylated	25 (56.8%)	79 (64.2%)	
Tumor location			
Frontal	13 (29.5%)	41 (32.5%)	0.926
Temporal	16 (36.4%)	40 (31.7%)	
Insular	9 (20.5%)	22 (17.5%)	
Parietal	4 (9.1%)	16 (12.7%)	
Occipital	2 (4.5%)	7 (5.6%)	
TERT promoter			
Mutant	11 (37.5%)	49 (42.3%)	0.080
Wild	17 (62.5%)	35 (57.7%)	
Patterns of progression			
Local recurrence	24 (54.5%)	103 (81.7%)	**0.003**
Distant metastasis	9 (20.5%)	8 (6.3%)	
Subependymal spread	9 (20.5%)	11 (8.7%)	
Leptomeningeal dissemination	2 (4.5%)	4 (3.2%)	

*T1CE, T1 weighted contrast-enhancing images; KPS, Karnofsky Performance Status score; GTR, gross total removal of tumor; MGMT, O-6-methylguanine DNA methyltransferase. Bold: significant*.

### The Incidence of Discordant T2/FLAIR and T1CE Images for GBM

We observed the dynamic change discordance between T2/FLAIR and T1CE in a small subgroup of patients with GBM (44/170, 25.9%). The abnormal finding of T2/FLAIR was prior to T1CE in most cases (37/44, 84.1%), and the four of remaining patients received re-operation before the new or constantly enlarged T1CE lesions emerged, and the other three patients were only found T2/FLAIR abnormal space-occupying lesions without enhancement till the last follow-up.

The median time-span between T2/FLAIR and T1CE indicated progression was 119.5 days (ranging 57 days -unreached). In the discordant patients, two developed leptomeningeal dissemination (2/44, 4.5%), nine presented subependymal spread (9/44, 20.5%), nine showed distant intracranial metastases (9/44, 20.5%), and the remnant 24 patients suffered *in situ* recurrences (54.5%). Generally speaking, nearly half of discordant patients were observed with non-local tumor progression (20/44, 45.5%), while in an accordant subgroup, only 18.3% of patients suffered from non-local progression (23/126, *p* < 0.001; Table 1). Therefore, newly emerged non-CE lesions, especially for these distant from the primary tumor cavity, should be cast more attention because T2/FLAIR abnormal lesions could still be the sign of GBM progression.

A total of 96 patients achieved gross total removal of tumor (GTR), and in the discordant subgroups, the GTR rate was higher than accordant patients (32/44, 72.7% for discordant and 64/126, 50.8% for accordant, respectively, *p* = 0.012). In addition, the preoperative status was better in discordant than the accordant group (preoperative Karnofsky Performance Status [KPS] score >70: 36 of 44 (81.8%) patients for discordant and 81 of 126 (64.3%) patients for accordant, *p* = 0.031). The phenomenon informed us that the discordant subgroup achieved better local tumor control and resulted in a lower incidence of local progression (Table 1). While the mean age, gender distribution, preoperative KPS score, mean tumor volume, the incidence of ventricle infringement, MGMT promoter status, EFGR amplification, tumor location, and TERT promoter status was not different between discordant and accordant patients (Table 1).

### Radiological and Pathological Finding

Distinguishing treatment-induced response, such as pseudoprogression and radionecrosis, from true progression is of utmost importance for subsequent clinical decision-making and prognosis assessment. In the whole discordant subgroup, eleven performed advanced imaging checks, such as MR spectrum (MRS), perfusion-weighted MRI (PWI), PET, or combined. Significantly increased choline (Cho)/N-acetyl-aspartate (NAA) ratio, relative cerebral blood volume (rCBV), and high glucose uptake were observed in these nine patients (9/11, 81.8%) while the other two showed mild-to-moderate perfusion and metabolic transformation.

**Figure 2:**
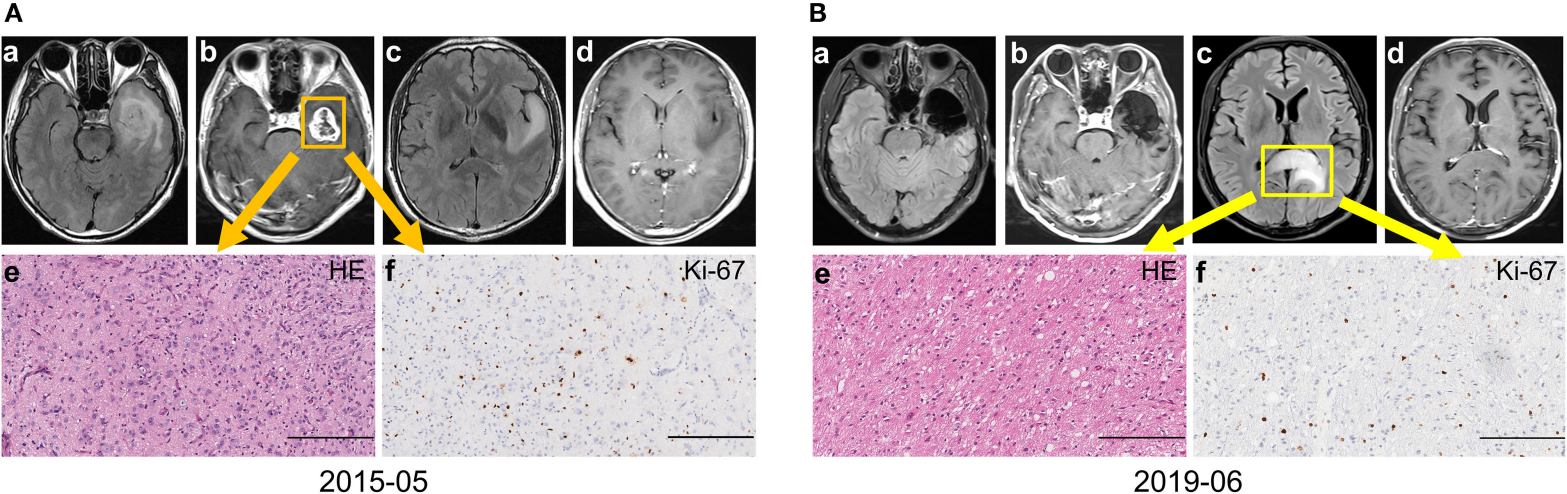
A representative case for the discordant subgroup with distant intracranial metastasis. **(A)** The patient presented unbearable headache with contrast-enhancing (CE) lesion on left temporal (a–d). Craniotomy was performed and the tumor was totally removed. The final diagnose was primary GBM, IDH wild-type, WHO grade 4 (e–f). **(B)** Forty-nine months after the operation, a non-CE lesion on the splenium of the corpus callosum with space-occupying effect (a–d) was suspected tumor progression. The final pathology diagnosis was GBM, IDH wild-type, WHO grade 4 (e–f) based on the WHO 2021 brain tumor classification system (Bars, 200 μm). GBM, glioblastoma; IDH, isocitrate dehydrogenase.

Eleven patients in the discordant subgroup (three of them performed advanced imaging check, and seven of them were local recurrence) accepted reoperation, and eight of them were reported with GBM (all accepted re-operation till the tumor evolved into CE). The other three without enhancement were histologically confirmed astrocytoma with anaplastic characteristics (no obvious microvascular proliferation or necrosis were observed though this diagnosis should be refined as GBM based on the 2021 WHO brain tumor classification examples in Figure 2), and all of them were distant metastasis. Therefore, highly aggressive GBM could transform into entities with histologically indicated gentle tumor behavior. This phenomenon was rare and has a close relationship with the distant non-CE lesion. Though most non-CE lesions evolved to CE lesions eventually, physicians should be aware that non-CE lesions might be the early sign of tumor progression for GBM, especially for distant lesions.

### Similar Prognosis Between Discordant and Accordant Patients

**Figure 3:**
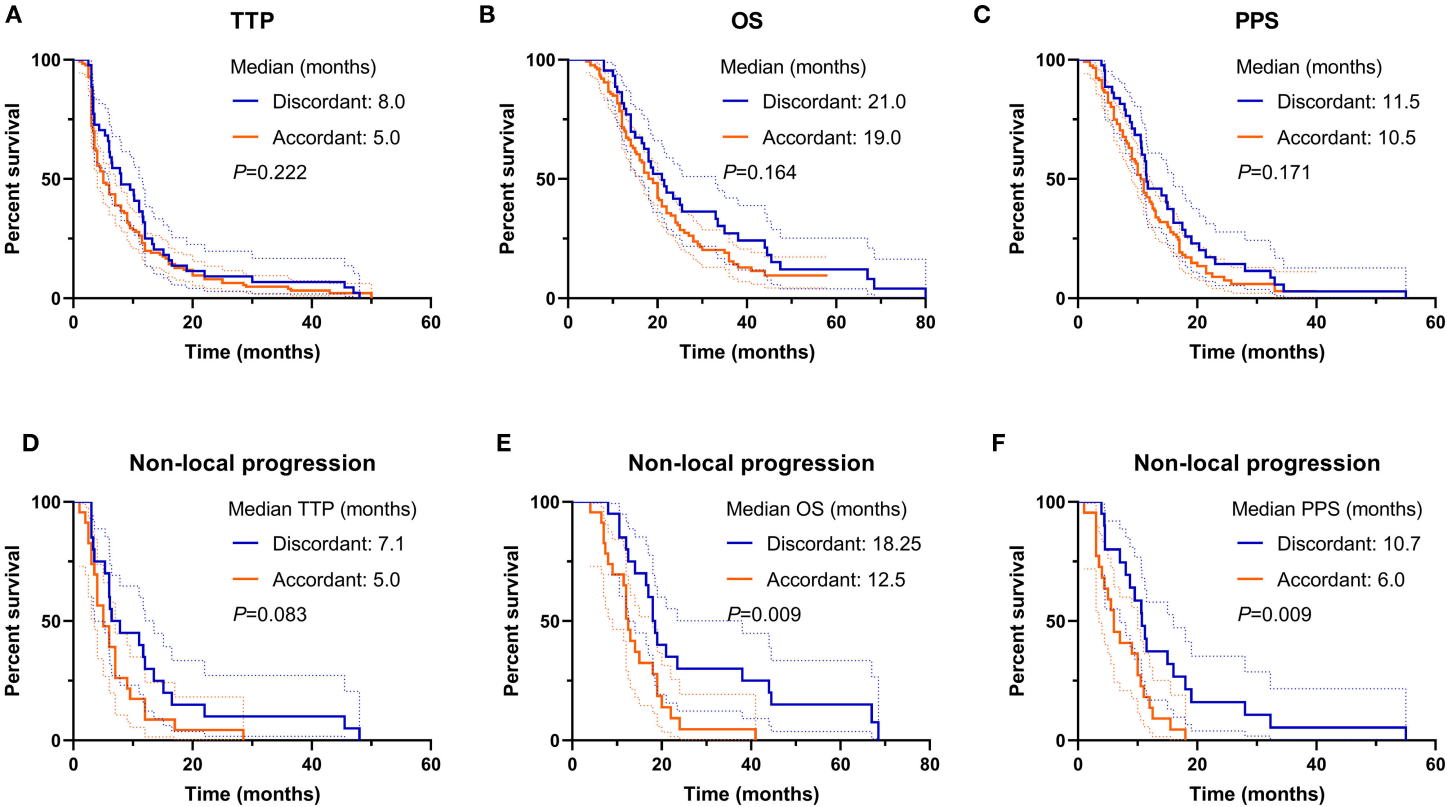
Prognosis comparison between T2/FLAIR and T1CE discordant and accordant patients. **(A–C)** The TTP, OS, and PPS were not different between the two subgroups. **(D–F)** For non-local progression patients, the OS and PPS in discordant subgroup were favorable than accordant subgroup, but not for the TTP. TTP, time to progression; OS, overall survival; PPS, post-progression survival.

For the whole cohort, the median TTP, OS, and PPS were 6.0, 19.0, and 11.0 months, respectively. The comparison of TTP, OS, and PPS between discordant and accordant patients was not significant (discordant vs. accordant, median TTP: 8.0 vs. 5.0 months, *p* = 0.222, Figure 3A; median OS: 21.0 vs. 19.0 months, *p* = 0.164, Figure 3B; median PPS: 11.5 vs. 10.5 months, *p* = 0.171, Figure 3C). The results demonstrated that though the discordant subgroup initially presented less aggressive tumor behavior with non-CE lesions, they progressed as fast as the CE lesions in accordant patients and harbored a lethal prognosis.

More discordant patients suffered from non-local tumor progression, thus simple comparison between discordant and accordant subgroups might overlook some critical information. Survival comparison demonstrated that the prognosis of non-local progression patients in the discordant subgroup was superior to accordant patients (median TTP: 7.1 vs. 5.0 months, *p* = 0.083, Figure 3D; median OS: 18.25 vs. 12.5 months, *p* = 0.009, Figure 3E; median PPS: 10.7 vs. 6.0 months, *p* = 0.009, Figure 3F). This result implied that the occurrence of tumor dissemination for discordant patients might be later due to better local tumor control, and disseminated nCE tumor cells might harbor a relatively gentle tumor behavior in discordant patients.

## Discussion

The IDH wild-type *de novo* GBM is one of the most lethal malignancies that embraces highly molecular, temporospatial, and radiological intratumoral and intertumoral heterogeneity (10). The RANO and modified RANO criteria for GBM request newly emerged or constantly enlarged CE lesions as the basic prerequisite to defining progression, regardless of T2/FLAIR (14–16). Our longitudinal observation demonstrated that extra attention should be paid to the dynamic change of T2/FLAIR, which might be the early sign of progression, especially for non-local progression GBMs.

Typically, GBM is highly aggressive, infiltrative, and invasive brain malignancy with prominent blood-brain barrier disruption (10). The canonical manifestation on radiology is pronounced CE lesions with surrounding T2/FLAIR abnormity areas (23). However, the inherently high heterogeneity renders neither all GBMs nor the whole body of GBMs is aggressive and destructive enough that could be reflected by CE MRI. Not only a minority of GBMs were non-CE but the ratio of nCE/CE in the GBMs varied significantly (24, 25). Both the CE and T2/FLAIR abnormal regions are enriched with malignant tumor cells that could reproduce and propagate rapidly (7, 26, 27). Contemporarily, no assessment criterion focusing on GBM was established according to radiological alteration of T2/FLAIR, neither treatment effect assessment for Bevacizumab-naïve patients nor progression surveillance. The extent of resection (EOR) of GBM is based on the percentage of CETs removed or the accurate resident volume of enhancement, regardless of T2/FLAIR. Complete response, partial response, stable disease, or progression after treatment for GBM of RANO was defined by dynamic alteration CE lesions for patients who did not receive antiangiogenic therapy (14, 28, 29). Studies reported the sensitivity of FLAIR signal increase prior to enhancement indicated *in situ* progression ranged from 34 to 75% (27, 30–33), higher than ours, which might be accountable due to the difference in inclusion criteria, GTR, and ventricle infringement rate in our study. Our study firstly reported the incidence of T2-FLAIR discordance in dissemination monitoring and revealed its poor clinical outcome.

Traditionally, multifocal or multicentric GBM refers to the concept that synchronous multiple lesions at diagnosis, and the former was defined as multiple CE lesions embedded in a relatively large area with abnormal T2/FLAIR-weighted signal while the latter usually invading different hemisphere or lobe devoid of connection. Furthermore, multiple GBMs occurred at a distinct time without connection on MRI were also viewed as multicentric GBM (2, 34). However, these definitions were established based on the CE lesions with/without well-demarcated margins. Lasocki et al. firstly reported that the incidence of multicentric nCE lesions of GBM was 6% (9/151) at preoperative diagnosis, and the survival was much worse than patients without multicentric non-enhancement lesions. In four patients with follow-up MRIs, all developed enhancement and necrosis within 1 year (11). This phenomenon informed us that nCE lesions distant from the dominant lesion could be the reason for disease progression and treatment failure, and much more attention should be attached to the nCE lesions because they could not only be multicentric GBMs at diagnosis but at recurrence. Future response assessment criteria should incorporate the dynamic change of T2/FLAIR especially distant signal alteration to monitor GBM *in situ* recurrence or intracranial dissemination.

Local recurrence dominates the patterns of GBM progression (ranging from 60 to 85%) (12). The standard care of GBM demands radiotherapy to eliminate the residual tumor cells and postpone tumor recurrence. Demyelination caused by radiation in target volume could lead to evident T2/FLAIR abnormity (16). At the very early stage of progression, tumor-related edema or infiltration effect was quite obscure and totally covered by demyelination on T2/FLAIR images, but even tiny CE lesion on MRI could be extraordinarily conspicuous on T1CE images. Thus, for local recurrence and leptomeningeal dissemination monitoring, the newly emerged or constantly enlarged CE lesions could be more sensitive for recurrence predicting. Tumor cells could also migrate into areas out of beam target or the low dose of the target where demyelination was less pronounced. Tumor cells from nCE areas of initially dominant bulk could form new non-CE lesions out of the original target zone, or at the very early stage of new lesion forming, the transformation of T2/FLAIR was prior to T1CE images. Our results showed that 25.9% of patients could be found with an nCE lesion that eventually developed into CE lesions with necrosis, and three of them were pathologically confirmed as nCE GBM. Berzero et al. reported that histological grading was important for IDH wild-type glioma prognosis assessment (35), and our result confirmed this result in non-local progressed discordant patients. Thus, T2/FLAIR should be added to determine dissemination and predict prognosis for GBM, especially in the very early stage.

Currently, the concept of supratotal maximal resection (SMR), which is termed as total removal of both CE and T2/FLAIR abnormal regions for eligible patients, has raised great interest in neurosurgeons for GBM treatment. Some retrospective studies demonstrated that patients with GBM might derive a survival benefit from SMR while some were not (36–44). GBMs appropriate for SMR are commonly located on the prefrontal lobe, where a high percentage of GBMs was glioma-CpG island methylator phenotype (G-CIMP) subtype and MGMT promoter methylated (45, 46). The evidence level of SMR for GBM could never be equal to a multicenter, prospective trial due to the impractical nature of exploring the impact of EOR on survival. Though some results were contradictory, we still could not entirely deny the benefit yielded from SMR. This is the initial concern for T2/FLAIR abnormity for GBM, and we believe this would be inspirable and enlightened.

Multiple recurrences in GBM indicated a more aggressive and invasive tumor behavior and portended inferior prognosis without exception. There still lacks efficient and effective treatment modalities for GBM dissemination control. Some polite studies revealed that stereotactic radiosurgery (SRS) might be helpful for a single, small lesion with the satisfactory disease control (19, 47). For eligible patients, SRS might be an ideal choice to alleviate suffering and prolong survival.

Limitations do exist due to the nature of retrospective studies within a single institute. Acquisition of consecutive MRI data every 2–3 months in a large cohort of patients was quite difficult. Different medical centers for follow-up, poor compliance, and financial problems might be the major reasons that impede us from sufficient data. Till now, this is nevertheless one of the largest cohorts of studies with a series of MRI to dynamic monitor treatment response. Another limitation lies in that pathology confirmation was only achieved in a small part of patients. Furthermore, distinguishing peritumor edema from true non-enhancing tumors was difficult under certain circumstances. These limitations could not cover up the meaningful finding of this study, and neuro-oncologist should shed more light on the dynamic image change during follow-up, not only T1WI CE but T2/FLAIR images.

## Conclusion

T2/FLAIR abnormity could be the early sign of GBM progression, especially for newly emerged lesions distant from the primary tumor cavity. The subsequent modified GBM assessment criterion should incorporate the T2/FLAIR information for disease monitoring, and physicians should cast more attention on the dynamic change of T2/FLAIR images for progression evaluation and subsequent clinical decision-making.

## Data Availability Statement

The original contributions presented in the study are included in the article/supplementary material, further inquiries can be directed to the corresponding authors.

## Ethics Statement

The studies involving human participants were reviewed and approved by Capital Medical University, Beijing. The patients/participants provided their written informed consent to participate in this study.

## Author Contributions

ML, XR, HJ, CY, YC, and SL contributed to literature search, study design, data collection, data interpretation, and writing. HC, SS, and XR contributed to MRI analysis. ML, SS, and CY contributed to data analysis. All authors contributed to the article and approved the submitted version.

## Funding

This study was supported by the National Natural Science Foundation of China (81571632&81771309).

## Conflict of Interest

The authors declare that the research was conducted in the absence of any commercial or financial relationships that could be construed as a potential conflict of interest.

## Publisher's Note

All claims expressed in this article are solely those of the authors and do not necessarily represent those of their affiliated organizations, or those of the publisher, the editors and the reviewers. Any product that may be evaluated in this article, or claim that may be made by its manufacturer, is not guaranteed or endorsed by the publisher.

## Abbreviations

GBM: glioblastoma
IDH: isocitrate dehydrogenase
MGMT: O^6^-methylguanine-DNA methyltransferase
KPS: Karnofsky Performance Status score
GTR: gross total removal of tumor
SMR: supratotal maximal resection
TTP: time to progression
OS: overall survival
PPS: post-progression survival.
